# Hospital Meetings, &c.

**Published:** 1897-02-20

**Authors:** 


### HOSPITAL MEETINGS, &C.

### UNIVERSITY COLLEGE HOSPITAL.

The receipts at the festival dinner in aid of the above hospital, which was held on Wednesday, the 10th inst., at the Hotel Cecil, the Lord Mayor presiding, reached the highest point yet touched by gatherings of a similar character in aid of University College Hospital. Within a few pounds of £5,000 was collected, the nearest approach to that sum in the past being £4,850, collected at the festival dinner in 1894, under the presidency of the Hon. Mr. Justice Charles. In only one other year has the dinner, or the special appeal which the hospital on several occasions has substituted for the dinner, reached over £4,000, viz., in connection with a public meeting held in 1884 at the Mansion House under the presidency of the then Lord Mayor.

The Lord Mayor, in proposing the toast of the "Prince of Wales (vice-patron), the Princess of Wales, and the Rest of the Royal Family," expressed the hope that the noble work of helping the hospitals inaugurated by the Prince of Wales might prove a success. His Royal Highness had taken up that noble cause with all the devotion of which he was capable, and his efforts he (the speaker) was sure would be loyally seconded by a devoted English people. (Applause.)

In proposing "Prosperity to the Hospital," the Lord Mayor made a very telling appeal on its behalf. H.R.H. the Prince of Wales had asked him whether he thought his noble scheme would interfere with the Indian Famine Fund, and he (the speaker) had replied that he did not think it would in the slightest. The call upon the charity of English hearts was never too big nor too great and the English purse was inexhaustible. People said that the management of the hospitals left much to be desired, to which he would reply that there was no community which during the year did not experience losses, but at the end of the year one looked at the balance and not at the varying difficulties which one had had to encounter. The best that any institution could look to obtain was a balance on the right side, and the hospitals of London had a balance on the right side—not in money, Heaven forbid, but a balance of noble and excellent work done for the community. "Never mind" he continued, "the management—that can be mended—but aid the hospitals, for we cannot do without their good work." (Applause.)

### ST. MARK'S HOSPITAL FOR FISTULA.

The annual meeting of the St. Mark's Hospital for Fistula, City Road, was held at the Mansion House on Thursday (11th inst.), the Lord Mayor in the chair. The business was of a formal character and consisted of the adoption of the report and the passing of votes of thanks to the various officers connected with the institution. The report stated that in January, 1896, two of the four wards were opened for patients and during the year 237 in and 841 out-patients were treated. The ordinary income amounted to £1,798 and legacies to £1,667, while the ordinary expenditure reached £3,307, in addition to which a sum of £395 was spent on furniture.

### ROYAL NATIONAL HOSPITAL FOR CONSUMPTION.

The annual general meeting of the governors of this hospital was held on Thursday, 11th inst., at the offices, 34, Craven Street, W.C., Dr. J. S. Coghill in the chair. The report stated that the board of management had been enabled to keep every bed occupied, and the number of in-patients under treatment during 1896 had exceeded the number in any previous year. The patients treated amounted to 810, of whom 63 attended also as out-patients while awaiting their turn for admission. In view of the large number of patients on the books, many of whom had been compelled to wait three months or more before a vacancy allowed of their being taken in, the board felt that the time had now arrived when they must endeavour to enlarge the accommodation at the hospital. It was the earnest desire of the board that the necessary funds for the cost of a new block of three houses should be received in this the sixtieth year of Her Majesty's reign. Each of the three houses was estimated to cost about £2,000. Princess Henry of Battenberg had consented to lay the foundation-stone of the new block in the latter part of July. The total receipts amounted to £11,831, and the expenditure to £12,031. The report was adopted on the motion of the Chairman, seconded by Mr. N. F. Horne (deputy-chairman of the hospital). Mr. P. B. Burgoyne was elected treasurer of the hospital.

